# Differences in police, ambulance, and emergency department reporting of traffic injuries on Karachi-Hala road, Pakistan

**DOI:** 10.1186/1756-0500-4-75

**Published:** 2011-03-22

**Authors:** Junaid A Bhatti, Junaid A Razzak, Emmanuel Lagarde, Louis-Rachid Salmi

**Affiliations:** 1 Équipe Avenir « Prévention et Prise en Charge des Traumatismes », Institut National de la Santé et de la Recherche Médicale Unité 897 (INSERM U897), Bordeaux, France; 2 Department of Emergency Medicine, The Aga Khan University, Karachi, Pakistan; 3 Douglas Mental Health University Institute, McGill University, Montreal, Canada; 4 Institut de Santé Publique, d'Épidémiologie et de Développement (ISPED), Université Bordeaux Segalen, Bordeaux, France; 5 Service d'information médicale, Centre Hospitalier Universitaire de Bordeaux, Bordeaux, France

**Keywords:** Highway, injury severity, surveillance, traffic accident

## Abstract

**Background:**

Research undertaken in developing countries has assessed discrepancies in police reporting of Road Traffic Injury (RTI) for urban settings only. The objective of this study was to assess differences in RTI reporting across police, ambulance, and hospital Emergency Department (ED) datasets on an interurban road section in Pakistan.

**Methods:**

The study setting was the 196-km long Karachi-Hala road section. RTIs reported to the police, Edhi Ambulance Service (EAS), and five hospital EDs in Karachi during 2008 (Jan to Dec) were compared in terms of road user involved (pedestrians, motorcyclists, four-wheeled vehicle occupants) and outcome (died or injured). Further, records from these data were matched to assess ascertainment of traffic injuries and deaths by the three datasets.

**Results:**

A total of 143 RTIs were reported to the police, 531 to EAS, and 661 to hospital EDs. Fatality per hundred traffic injuries was twice as high in police records (19 per 100 RTIs) than in ambulance (10 per 100 RTIs) and hospital ED records (9 per 100 RTIs). Pedestrian and motorcyclist involvement per hundred traffic injuries was lower in police records (8 per 100 RTIs) than in ambulance (17 per 100 RTIs) and hospital ED records (43 per 100 RTIs). Of the 119 deaths independently identified after matching, police recorded 22.6%, EAS 46.2%, and hospital ED 50.4%. Similarly, police data accounted for 10.6%, EAS 43.5%, and hospital ED 54.9% of the 1 095 independently identified injured patients.

**Conclusions:**

Police reporting, particularly of non-fatal RTIs and those involving vulnerable road users, should be improved in Pakistan.

## Background

Pakistan, located at the junction of Middle-East, South-East, and Central Asia, is the sixth most populous nation of the world [1]. According to transport authorities, approximately 1.4 million Road Traffic Crashes (RTCs) occurred in Pakistan in 1999, resulting in over 7 000 fatalities [2,3]. Two independent population-based surveys estimated the incidence of Road Traffic Injuries (RTIs) to be around 15 to 17 per 1 000 persons per year [4,5]. These injuries contributed significantly to the workload in hospitals, leading to direct costs to the Pakistani economy of over one billion US dollars [6,7].

Road transport, in Pakistan as in most countries, is the backbone of the economy. Interurban roads are distinguished from rural roads by higher traffic counts and speeds. For instance, the strategic interurban road network of Pakistan, which is approximately 8 000 km long, carries more than 80% of inland passenger and freight traffic [2,8]. Published statistics showed that these road sections accounted for a high proportion of traffic fatalities (27%) although they accounted for only 4% of the entire network [9]. Higher speeds, presence of vulnerable road users, and complex road traffic conditions can explain this high fatality ratio, but no comparison indicators were available for such road sections [10].

Because of geographical distances and complexity of trauma care in interurban settings, police records remain, to date, the most used source for evaluating interurban traffic safety [11,12]. The use of these statistics, however, can lead to underestimation of RTI burden in Low and Middle Income Countries (LMICs) like Pakistan [13]. A recent World Health Organization (WHO) report showed that actual traffic fatalities could be 4 to 10 times higher than the official statistics in Pakistan [14]. A previous study in Karachi showed that police records accounted for only 56% of traffic fatalities and 4% of severe injuries [15]. No notable research has been carried out to compare the differences in injury reporting by linking different datasets for interurban road settings in Pakistan [13,14]. The World Bank reported that interventions with proven effectiveness exist but their implementations are impeded by the lack of documenting specific disease burden in LMICs [16]. The objective of this study was to assess differences in traffic injury reporting in terms of road user groups and outcome, between police, ambulance, and hospital Emergency Department (ED) datasets for an interurban road section in Pakistan. Further, these datasets were linked to assess variations in traffic fatality and injury per vehicle kilometre travelled on the road section.

## Methods

The study setting was the 196-km-long Karachi-Hala road section (km 16 to km 212 from Karachi centre), for which the three RTI databases were available. This is a four-lane highway, two lanes in each direction [8]. The lanes are separated by a ground surface, but there are no physical barriers. Traffic counts vary between 16 356 to 24 707 vehicles per day on this section [17]. These high traffic counts are related to the economic activity in Karachi, the most populous city of Pakistan, accounting for 70% of government's trade and industry-related revenue [18]. In this retrospective study, characteristics, such as outcome and user category, of traffic injury patients reported to highway police, ambulance service, and hospital ED from January to December 2008 were compared among the three databases. Data on crash characteristics were too scarce to be compared.

### Case definitions

A crash was defined as any event where a motorized vehicle, including motorcycles, was involved in a collision with another vehicle, road user, or other obstacle, and reported in either of the police, ambulance, and hospital ED datasets [13,15]. RTI was defined as any person incurring a physical injury as a result of a crash reported to any of the above datasets [13,15].

### Police data

Since 2004, the National Highway & Motorway Police (NHMP) has been enforcing traffic rules on this road section. Administratively, this section is considered as Sector I of South-Zone of NHMP and is divided further in four 46 to 51 km-long beats: beat 35 (km 16 to 62 km), beat 34 (63 to 114 km), beat 33 (115 to 162 km), and beat 32 (163 to 212 km). NHMP deploys on each beat four motor vehicles and four patrolling officers per eight-hour shift [19].

For every crash, a standard accident analysis report is filed during the first 24 hours by the attending NHMP officer [20]. A copy of this report is kept in the NHMP regional office. Details on the crash and those involved are recorded on a separate accident register. From these reports and registers, information was extracted on time, date, location of crash, and whether it was fatal, involved injury, or was without injury. We also extracted information on name, age, gender, outcome (dead; transported to hospital; and not transported to hospital), and, if transported, name of the hospital.

### Ambulance data

Ambulance records were obtained from Edhi Ambulance Service (EAS) logbooks. EAS is the largest private philanthropic ambulance service in the world [21]. Since 1973, the EAS has been progressively increasing its ambulance posts from main Pakistani cities to the important highways in Pakistan [22,23]. For transporting injured patients, EAS has established six ambulance posts, mostly near main towns on Karachi-Hala road section: 1) Sohrab Goth (12 km from Karachi centre), 2) Karachi toll plaza (km 28), 3) Edhi centre (km 56), 4) Nooriabad (km 94), 5) Hala Naka (km 160), and 6) Hala city (km 212). This service is freely available to injured patients, and funds are raised by transporting other patients. In most cases, ambulances are only staffed by the driver. A clerk at the post can come with the driver if he thinks this is justified, for instance, crashes with multiple patients. The ambulance communicates with the emergency post through a wireless system or cell phone.

RTI patients or bystanders can contact EAS using the free emergency-access number 115, which connects them to the main city centre [21]. Information is then transmitted by wireless or cell phone to nearby posts, which finally dispatches the ambulance(s). When reaching the scene, attendants separate injured from dead patients. Those severely injured are transported to the nearest hospital; preference is given to the government hospital if available. All information on the intervention, including crash location, injured patients identity and outcome, is then transmitted by wireless or telephone to the regional centre, which records the information in a central log book. We photocopied these log books from the regional centre at Karachi. Crash details such as date, time, location, and whether it was fatal or involved injury were extracted from these books. Similarly, road user details such as name, gender, age, user type (pedestrian, motorcycle rider, or vehicle occupant), and outcome (died, including whether the person died at crash scene, during transport, or at hospital ED; injured and transported, including hospital taken to; injured and not transported) were extracted from these log books [21].

### Hospital records

The Road Traffic Injury Research & Prevention Centre (RTIRP) at the Jinnah Post Graduate Medical Centre (JPMC) has systematically collected standard forms since September 2006 [24], information on RTI patients presenting at the hospital ED of the five largest teaching hospitals in Karachi: 1) JPMC, 2) Abbasi Shaheed Hospital, 3) Civil Hospital Karachi, 4) Liaqat National Hospital, and 5) The Aga Khan University Hospital. Details on their data collection methods are described elsewhere [24,25].

This dataset includes information on the crash date, time, and location, and patient's name, age, gender, road user type (pedestrian, motorcycle rider, or vehicle occupant). Further information on whether the patient was wearing a helmet or seat belt was available. The New Injury Severity Scores (NISS, range 1 to 75) [26], and outcome (discharged, admitted/referred, or died) of patients were recorded during their stay in the hospital ED. Information on RTI patients involved in crashes on selected road section was extracted from this dataset.

### Analysis

All information was recorded in Excel^® ^spreadsheets. We compared percentages for crash and injury patient characteristics across the three datasets. For the hospital ED dataset, we described outcome for the following NISS categories: minor injury, scores ranging from 1 to 3; major injury, scores ranging from 4 to 8; and severe injury, scores above 9 [26]. Same records present in two or more datasets were matched using crash date and time, name, age, and gender of RTI patients. For matched records, we identified differences in reported outcome. To estimate total fatalities, a person reported injured in police statistics, but dead in ambulance data was considered as dead. The number of unique deaths and injuries were then assessed after removing duplicates of records appearing in two or more datasets. Ascertainment rate for police, ambulance, and hospital ED records, as compared to these total fatalities and injuries, were computed [27]. Capture-recapture methods were not used to estimate road burden, because RTIs away from Karachi might not have the same probability of being captured in the hospital ED dataset, thus violating one of the basic assumptions of the method [15]. The unique records and traffic counts from highway authority were used to compute overall traffic fatality and injury rates per vehicle kilometre in 2008 for this road section [17]. Considering that there would be missing information for variables used in linking datasets, we carried a secondary analysis considering situations where at least one of the variables could be matched.

### Ethical approval

All the police, ambulance, and hospital ED data used in this study were publicly accessible and analyses were conducted with approval from the respective institutions. Furthermore, this manuscript did not permit identification of any RTI patient.

## Results

### Patient characteristics

In 2008, 143 RTIs were reported to the police, 531 to EAS, and 661 to hospital ED. Names were available for 67.1% (n = 96) of police and 78.0% (n = 414) of EAS reported injury patients (Table 1). Information on age was available for 74.1% (n = 106) of police and 67.6% (n = 359) of EAS reported injury patients. Few records in the hospital ED dataset were without names (n = 13) or age (n = 5). The most injured patients in the three datasets were aged 16-45 years: 61.5% (n = 88) in police, 55.0% (n = 292) in EAS, and 78.1% (n = 516) in hospital ED. Males accounted for a majority of injuries, 92.1% (n = 609) of injured patients in hospital ED.

**Table 1:** Traffic injuries reported to police, ambulance, and hospital ED on Karachi-Hala road section (2008)

	Police		Ambulance		Hospital ED	
			
	n	%	n	%	n	%
Road traffic crash						
- Fatal	19	44.1	37	14.5	47	10.4
- Not fatal	24	55.8	184	72.2	372	82.9
- Unknown	0	0.0	34	13.3	30	6.7
						
Road traffic injury						
- Deaths	27	18.8	55	10.4	60	9.1
- Transported to hospital	80	55.9	428	80.6	601	90.9
- Not transported to hospital	36	25.2	48	9.0	NA	
						
Name of patient available*						
- Yes	96	67.1	414	78.0	648	98.0
- No	47	32.9	117	22.0	13	2.0
						
Age* (y)						
- 0-15	14	9.8	34	6.4	62	9.4
- 16-45	88	61.5	292	55.0	516	78.1
- >45	4	2.8	33	6.2	78	11.8
- Unknown	37	25.9	172	32.4	5	0.7
						
Gender*						
- Male	93	65.0	364	68.5	609	92.1
- Female	12	8.4	78	14.7	52	7.9
- Unknown	38	26.6	89	16.8	0	0.0
						
Road user group						
- Pedestrian	5	3.5	40	7.5	83	12.7
- Motorcycle riders	6	4.2	49	9.2	203	30.6
- Four-wheeled vehicles' occupants	120	83.9	403	75.9	327	49.5
- Others	0	0.0	1	0.2	4	0.6
- Unknown	12	8.4	38	7.2	44	6.6
						

ED- Emergency department

NA - Not applicable

* Variables used for matching records across the three databases.

The proportion of pedestrians in police reported crashes was 3.5% (n = 5), whereas this was 7.5% (n = 40) in the EAS and 12.7% (n = 83) in the hospital ED. The proportion of motorcycle riders in police reported crashes were 4.2% (n = 6), whereas this was 9.2% (n = 49) in EAS and 30.6% (n = 203) in hospital ED. Occupants of four wheeled vehicles accounted for a majority of injuries in the three datasets: 83.9% (n = 120) in police, 75.9% (n = 403) in EAS, and 49.5% (n = 327) in hospital ED. In the hospital ED, only 13.6% (n = 21) of the 154 injury patients riding motorcycles were wearing helmets. Similarly, only 3.2% (n = 3) out of 93 four-wheeled vehicle occupants were wearing a seat belt at time of crash.

### Crash and injury outcome

In 2008, police reported 43 crashes, whereas 255 crashes were reported to EAS and 449 to hospital ED. One out of two police reported crashes (n = 19, 44.4%) was fatal, whereas this proportion was 14.5% (n = 37) for those reported to EAS, and 10.4% (n = 47) for hospital ED. No information on crash outcome was available in 13.3% of EAS reported crashes, and 6.7% of those reported to hospital ED. Over half (n = 80, 55.9%) of police-reported injured patients received hospital care; 50.0% (n = 40) of these patients were injured between km 16 and km 120 and treated in Karachi. RTIRP hospitals accounted for 17 of those treated in Karachi. Nearly one fifth (n = 27, 18.8%) of RTI patients reported in police records died, whereas this proportion was 10.4% (n = 55) for EAS and 9.1% (n = 60) for hospital ED reported patients (Table 1). One fourth (n = 36, 25.2%) of police reported injured patients were not transported to the hospital, whereas this was 9.0% (n = 48) for EAS reported patients.

Out of 661 patients presenting to hospital ED, 47.7% (n = 315) arrived by private means, whereas 43.0% (n = 284) arrived in ambulances. Police transported only four of these patients, and no information was available on the remaining 8.8% (n = 58) patients. NISS were available for 92.8% (n = 614) of 661 hospital ED patients; 34.2% (n = 210) had minor, 28.0% (n = 172) had major, and 37.8% (n = 232) had severe RTI. All those who were reported to have died had a severe RTI. Of 206 admitted patients, 5.8% (n = 12) had minor injuries and 24.8% (n = 51) had major injuries.

### Concordance between databases

**Figure 1:**
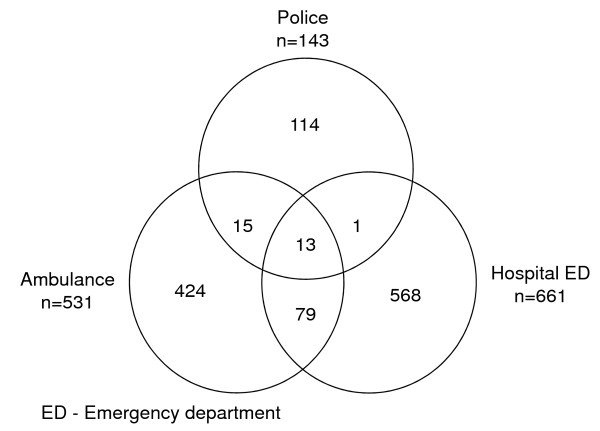
**Unique records of traffic injury patients reported to police, ambulance service, and hospital ED on Karachi-Hala road section in 2008 (n = 1 214)**.

A total of 108 patients were found in two or more datasets yielding 1 214 unique records from the three datasets (Figure 1). Of 143 police reported patients 20.3% (n = 29) were observed in other datasets; 19.5% (n = 28) in EAS and 9.8% (n = 14) in hospital ED. Of 531 EAS reported patients 20.2% (n = 107) were observed in other datasets; 5.3% (n = 28) in police and 17.3% (n = 92) in hospital ED. Of 661 hospital ED reported patients 14.1% (n = 93) were observed in other datasets; 2.1% (n = 14) in police and 13.9% (n = 92) in EAS.

Discrepancies were observed for outcome of injuries reported in police and ambulance records: four out of the 17 injured in police dataset were reported dead in EAS records. Similarly, one of eight injured in police records was reported dead in hospital ED records, and nine of 84 injured patients in EAS were reported dead in hospital ED records.

### Ascertainment of road fatalities and injuries

Based on matching, 119 patients died in 2008 on this interurban road section (Table 2); of these, police recorded 22.6% (n = 27), EAS 46.2% (n = 55), and hospital ED 50.4% (n = 60). Similarly, of 1 095 unique injured patients, police recorded 10.6% (n = 116), EAS 43.5% (n = 476), and hospital ED 54.9% (n = 601). Traffic fatality was 54 deaths and injuries were slightly over 500 per 109 vehicle kilometres travelled on this road section. Matching of nameless police and ambulance records, when any of the crash dates, time, age, and gender details was available, decreased the overall estimates by 4 deaths and 73 injuries. Corrected traffic fatality rate was 53 deaths and injuries 467 per 109 vehicle kilometres travelled on this road section.

**Table 2:** Ascertainment of police, ambulance, and hospital ED records for traffic fatalities and injuries on Karachi-Hala road section (n = 1 214)

Outcome	Police		Ambulance		Hospital ED		Total		Rate^†^
	n	%*	n	%*	n	%*	n	%	
									
									
Deaths	27	22.6	55	46.2	60	50.4	119	9.8	54.4
Injuries	116	10.6	476	43.5	601	54.9	1 095	91.2	500.4

ED- Emergency department

* Ascertainment rate; numbers of record divided by total for the given outcome.

† per 10^9 ^km travelled.

## Discussion

This study showed that crash and injury reports by police on this road section in a one-year period were several times less than ambulance and hospital ED data. Fatalities per hundred traffic injuries were twice as high in police records compared to ambulance and hospital records. On the contrary, pedestrian and motorcyclist involvement per hundred traffic injuries was twice as low in police records compared to ambulance and hospital records. Compared to overall estimated RTIs, police reported one of five traffic fatalities and one of ten traffic injuries on this road section.

Therefore, underreporting of traffic crash data by police in LMICs could be particularly high for non-urban road sections. Police accounted for only one of five traffic fatalities, compared to one out of two in Karachi [15]. This discrepancy could jeopardize resource allocation for traffic safety in these settings [28]. Police reporting is more reliable and complete in high-income countries (HICs), underscoring the need for improving reporting in LMICs like Pakistan, to better estimate and monitor traffic safety programs [13,29].

Reluctance of police to record non-fatal traffic injuries could explain the higher proportion of traffic fatalities in police than in both EAS and hospital ED records [15,19]. In Pakistan and many other LMICs, police performance is judged on few parameters [30]. Since RTCs are part of these parameters, higher traffic injury numbers could reflect poor enforcement. Documentation might be improved by implementing performance evaluation based on number of crashes in which the police intervened for public safety [30]. This might motivate police officers to report RTIs, thus improving identification of the high-risk groups and crash sites [14].

Police also reported fewer pedestrian and motorcyclist involvement per hundred traffic injuries than other sources. Firstly, it is likely that these injuries took place near built-up areas, so patients could have been transported by bystanders or ambulances directly to a hospital, without police intervention [14,31]. Secondly, these road users could belong to lower socioeconomic status, thus did not want to be involved in cumbersome and expensive legal procedures, and settled their issues without police [19]. Nevertheless, efforts are required to improve documentation of such road users to better design and implement effective crash prevention policies [32].

Limitations of secondary datasets such as ambulance or hospital ED for RTC prevention have been considered previously in Pakistan [33]. Availability of NISS was exceptional in this study, because of the existing RTI surveillance system [24]. It was observed that both EAS and hospital ED recorded the approximate location (nearby town, motel...), whereas police data included the km location of the crash site. Consequently, linking of these datasets identified a high crash and injury burden, but failed to identify high-risk crash sites. Moreover, seat-belt and helmet use was not reported in a majority of hospital ED patients, and not recorded at all in police data. This illustrated the need to improve police reporting of crash factors, information that could help in developing policies adapted to local settings [12].

Finally, this study may have some limitation regarding RTI estimates [33], because names were often missing in police and ambulance records. Some of these police and ambulance records could be matched on only one common variable, thus RTIs could be slightly overestimated. Nevertheless, corrected fatality and injury rates were higher than for a similar road in an HIC [34]. Moreover, fatality numbers could be even higher, because patients were not followed for over 30 days, as recommended by the WHO [14]. Furthermore, half of the police reported patients were injured away from Karachi and were transported to hospitals outside Karachi [31]. This shows that the ascertainment of police records could be even much lower than reported here [21].

## Conclusion

Interurban traffic crash burden appears to be several times higher in Pakistan than other HICs [34]. Police RTI documentation, particularly of non-fatal injuries and those involving vulnerable road users, should be improved in Pakistan [13,15,33]. Revising police performance evaluation, to account for number of traffic crashes in which the police intervened, might motivate officers to report RTIs [14,35]. Furthermore, a linked and comprehensive database would be useful to monitor and implement traffic safety interventions in Pakistan [15].

## Abbreviations

EAS: Edhi Ambulance Service; hospital ED: Emergency Department; HIC: High-Income Country; LMIC: Low and Middle Income Country; NHMP: National Highway & Motorway Police; NISS: New Injury Severity Scores; RTC: road traffic crash; RTI: road traffic injury; US: United States of America; WHO: World Health Organization.

## Competing interests

The authors declare that they have no competing interests.

## Authors' contributions

JAB, JAR, EL, LRS conceived of the study, and participated in its design and coordination. JAB and LRS performed statistical analyses. JAB wrote the first manuscript and LRS, EL, and JAR helped to finalize this manuscript. All authors read and approved the final manuscript.
